# Practical Pearls for Direct Detection of Live Treponema pallidum in Primary Syphilis Using a Phase-Contrast Microscope and USB Camera System: A Video-Based Technical Report

**DOI:** 10.7759/cureus.91702

**Published:** 2025-09-06

**Authors:** Yoshihiro Ono, Yoshiyuki Miyazawa, Seiji Arai, Yoshitaka Sekine

**Affiliations:** 1 Department of Urology and Venereology, Maebashi Primary Clinic, Maebashi, JPN; 2 Department of Urology, Gunma University School of Medicine, Maebashi, JPN

**Keywords:** chancre, phase-contrast microscopy, spirochete, syphilis, treponema pallidum, usb camera, video, wild-type

## Abstract

Syphilis cases are increasing in Japan, highlighting the urgent need for rapid and accurate diagnosis. In primary syphilis, serological tests may yield negative results, so direct microscopic observation of *Treponema pallidum* remains an important but often underused diagnostic method. We present a simple, widely accessible phase-contrast microscopy system, featuring a USB camera and monitor, enabling high-clarity, real-time observation of live wild-type treponemes directly from patient lesions. Specimens obtained from firm chancre rubs, mounted on sealed slides, were examined under oil immersion using 40× or 100× objective lenses. Treponemes were detectable within minutes, and serial imaging revealed striking morphological changes over time. Compared to dark-field microscopy, phase contrast provided superior image sharpness, digital recording capability, and greater value for patient education. Our video atlas is among the few contemporary records of motile wild-type *T. pallidum*, linking historical morphological studies to modern clinical practice. This inexpensive and reproducible method is well-suited for revitalizing microscopic diagnosis, training, and morphological research in syphilis.

## Introduction

The incidence of syphilis, including congenital and maternal cases, has increased in Japan since 2010 [1-3]. Early diagnosis and treatment are crucial to preventing further transmission and disease progression [4,5]. In primary syphilis, particularly in the earliest stages, serological tests may yield negative results [5,6]. Therefore, supplementary diagnostic methods such as molecular testing or direct microscopic detection of *Treponema pallidum* remain essential [6,7].

Dark-field microscopy is well established for detecting *T. pallidum* and is reported to have greater sensitivity than phase-contrast microscopy [5,7,8]. Nevertheless, phase-contrast microscopy remains valuable for observing the morphology and motility of live treponemes. In the 20th century, this technique was widely used for morphological studies of laboratory strains, such as the Nichols strain [9]. However, recent literature has emphasized serological and molecular testing, with limited focus on real-time microscopy [8,10,11]. As a result, many modern clinicians and laboratory technicians lack experience with direct treponemal visualization. Published videos or visual references of live wild-type *T. pallidum* from patient samples are virtually nonexistent.

To address this gap, we present a practical, accessible method for observing live *T. pallidum* using a phase-contrast microscope with a USB camera system. This system has proven effective in daily clinical practice by enabling real-time diagnosis, visual confirmation, and immediate treatment initiation within a single consultation.

## Technical report

Equipment

**Figure 1:**
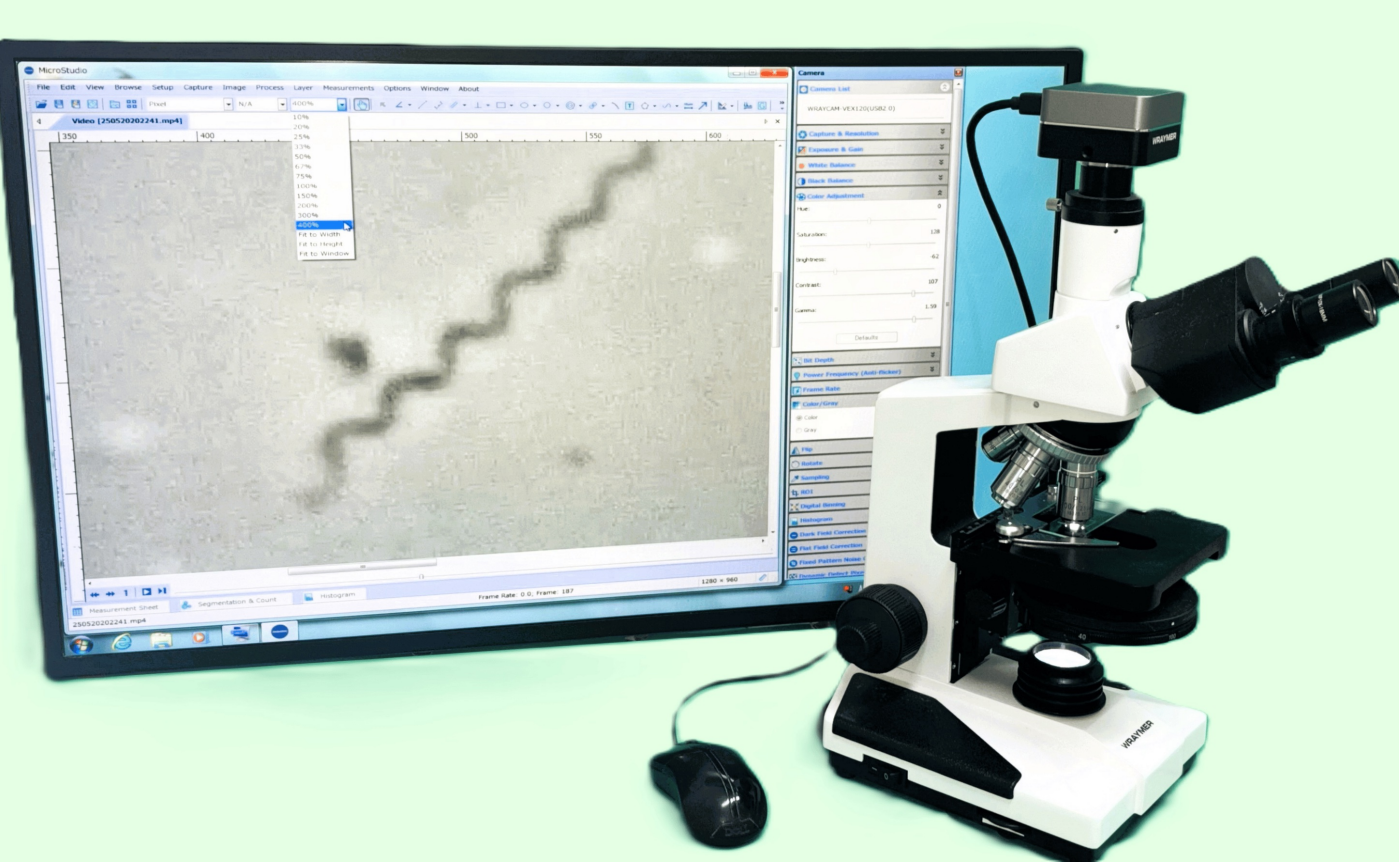
Phase-contrast microscopy and USB camera system The condenser and objective lens are for phase-contrast microscopes, and the USB camera captures the image without using an eyepiece lens. The monitor displays a *T. pallidum* image obtained with a 100× phase-contrast microscope objective lens, enlarged 400% using imaging software.

The observation system comprised a phase-contrast microscope, a USB camera, and built-in image-processing software (Figure 1). The image was displayed on a computer monitor, allowing adjustments of brightness, contrast, gamma correction, and switching between color and grayscale modes. In this study, we used a BX-2708TPHL microscope with a WRAYCAM-VEX120 USB camera (Wraymer Co., Ltd., Osaka, Japan). Similar systems are widely available and affordable.

Specimen preparation

The procedures for specimen preparation are shown in Video 1.

**Video 1:** Procedures from specimen collection to microscopic observation Sequential demonstration from chancre swabbing, dipping in saline, slide preparation, sealing, applying oil immersion, and placement under the phase-contrast microscope for examination.

**Figure 2:**
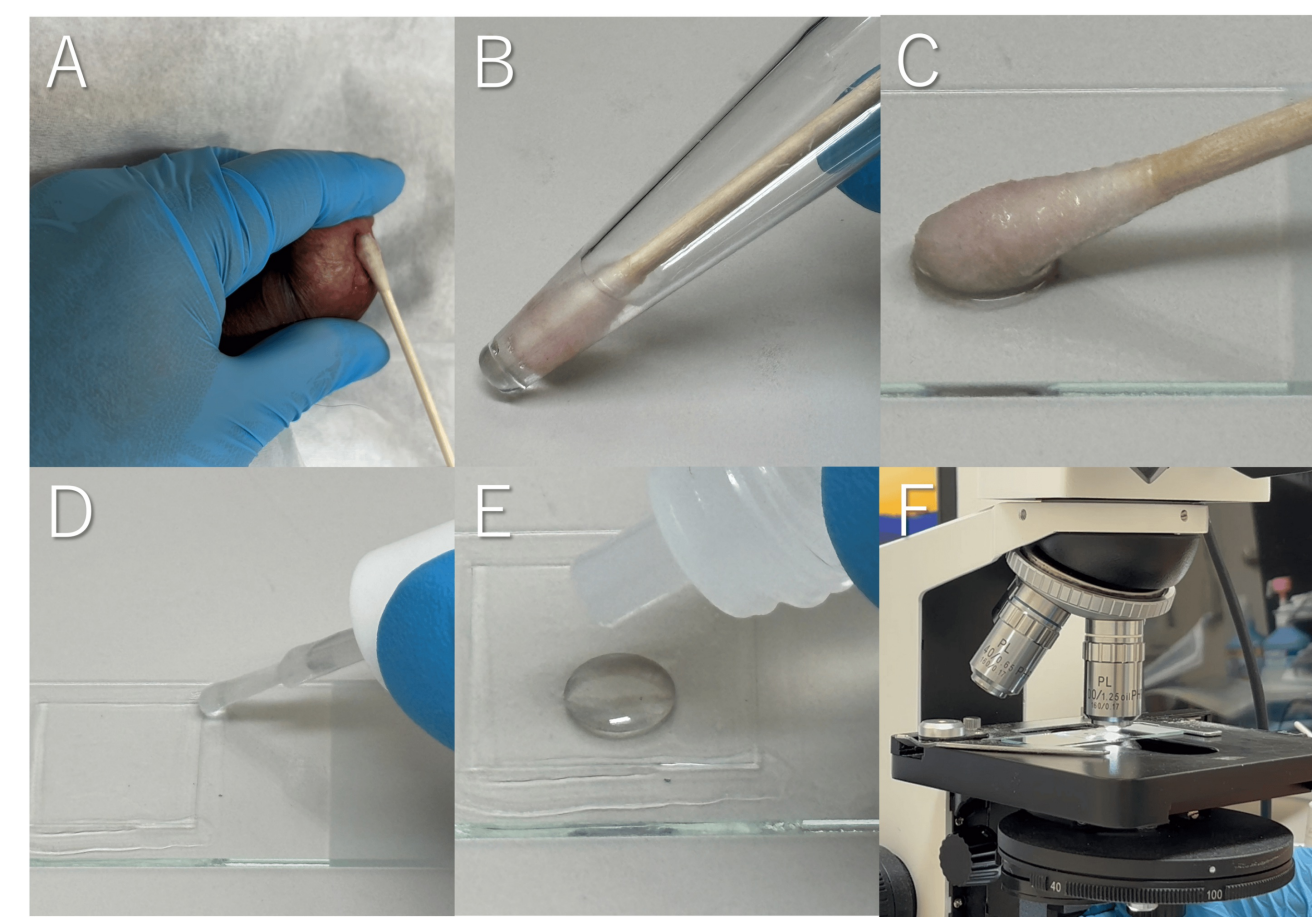
Procedures from specimen collection to microscopic observation Sequential steps including lesion swabbing (A), dipping in saline (B), slide preparation (C), sealing (D), applying oil (E), and placement under the phase-contrast microscope for examination (F).

The chancre surface was first cleaned to remove necrotic tissue and debris. Exudate was then collected by firmly rubbing the lesion with a cotton swab (Figure 2A), which was optionally pre-moistened with saline. The swab was dipped into saline (Figure 2B), and the material was smeared onto a glass slide (Figure 2C). A coverslip was placed over the specimen, lightly pressed, and the edges were sealed with nail enamel or another sealant (Figure 2D). Finally, immersion oil was applied (Figure 2E), and the slide was positioned on the microscope stage for examination (Figure 2F).

Observation

The initial search was conducted using a 40× objective lens, followed by a detailed assessment with a 100× oil immersion objective lens to evaluate morphology and motility. Image quality was enhanced by adjusting brightness, contrast, and gamma settings, and by switching to grayscale mode. The X-Y stage controls were used for systematic scanning of the slide, and while digital zoom allowed enlargement of the image, it did not improve resolution.

Results

The results are shown in Video 2.

**Video 2:** Atlas of the live syphilis spirochete T. pallidum Phase-contrast microscopic footage using a 100× objective lens, showing *T. pallidum* at different time points after specimen collection. Within the first four hours, the spirochetes exhibit vigorous rotation, active forward movement, and slightly irregular spiral forms. Over time, motility decreases, the spiral forms become partially flattened and wavy, and eventually, many organisms cease movement.

With sufficient familiarity, live treponemes could typically be detected within five minutes of specimen collection. In contrast, confirming the absence of *T. pallidum* required more prolonged scanning, often extending beyond 30 minutes, even for experienced observers.

**Figure 3:**
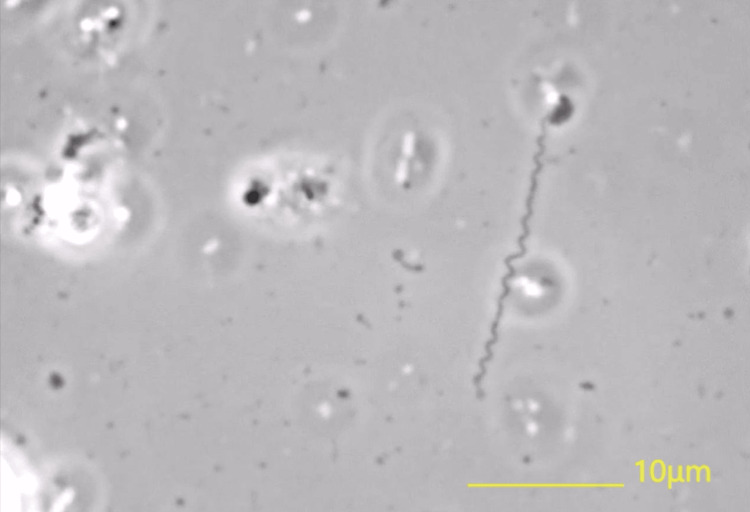
Atlas of the syphilis spirochete T. pallidum Phase-contrast microscopic image using a 100× objective lens, showing the characteristically irregular and slightly flattened spiral forms of *T. pallidum*, observed three hours after specimen collection. The patient was a 35-year-old man, and serological testing was positive (RPR: 4×, TPHA: 640×). RPR: rapid plasma reagin, TPHA: Treponema pallidum hemagglutination assay

Time-lapse observations demonstrated dynamic changes in morphology and motility: within four hours of collection, the organisms displayed vigorous rotation, active forward movement, and irregular spiral forms. By six hours, motility was reduced, and the organisms appeared more wavy or flattened. At around 12 hours, movement was minimal, and many, although not all, spirochetes had ceased activity. On rare occasions, slightly motile *T. pallidum* could still be observed even after 36 hours. Still images provided a morphological atlas (Figure 3), while accompanying videos documented these temporal changes in behavior and structure.

## Discussion

Direct microscopic observation of *T. pallidum* has a long tradition in clinical microbiology but has been overshadowed by modern serological and molecular methods [5-8]. While these tests offer excellent accuracy, they may fail to detect very early infections during the seronegative window period [5,6]. In such cases, direct visualization may contribute to more rapid diagnostic confirmation.

Dark-field microscopy remains the gold standard for detection, yet phase-contrast microscopy offers sharper structural visualization and better motility assessment [8,12,13]. In dentistry and oral surgery, phase-contrast microscopy is routinely used for real-time patient demonstrations, combining diagnosis and education in one encounter [12-15]. In contrast, syphilis diagnosis in hospital settings is often fragmented among physicians, technicians, and laboratories, potentially delaying results [8,16].

In our clinic, this system has been integrated into daily workflow, allowing specimen collection, microscopic examination, diagnosis, and patient education within a single visit, mirroring the efficiency seen in dental practice.

Although high-quality videos have been presented in basic experimental studies using cultured strains [17], very few modern videos exist of live wild-type *T. pallidum* [18,19]. Historical records capture only cultured strains, and live-motion footage was not preserved [9]. Our video atlas fills this gap, serving as a reference for clinicians who have never observed the organism alive.

Classical microscopic techniques, when combined with modern, affordable, and widely available tools such as USB cameras and imaging software, can now be performed more easily than ever. This approach should be reconsidered and re-evaluated for broader adoption in clinical practice, not only to improve diagnostic accuracy but also to preserve vital observational skills.

## Conclusions

Phase-contrast microscopy combined with a USB camera and standard imaging software provides an inexpensive, practical, and widely accessible method for directly observing *Treponema pallidum* in patient specimens. This system enables real-time visualization, supports rapid diagnosis, and facilitates patient education in routine clinical practice. Our video atlas offers one of the few modern references to live wild-type *T. pallidum*, helping to preserve essential microscopic diagnostic skills. The integration of this approach into daily workflow demonstrates its potential value for revitalizing direct treponemal detection and strengthening diagnostic capacity in an era dominated by serological and molecular testing.
